# Exploration of the quality of participation in an inclusive ultra-trail initiative for people with a disability: a qualitative ethnographic study

**DOI:** 10.3389/fspor.2026.1771945

**Published:** 2026-06-05

**Authors:** Raphaël Ouellet, Juliette Seroussi, Joanie Bédard, Jade Berthiaume, Éric Sévellec, François Routhier, Véronique H. Flamand, Krista L. Best

**Affiliations:** 1 Centre for Interdisciplinary Research in Rehabilitation and Social Integration, Centre Intégré Universitaire de Santé et de Services Sociaux de la Capitale-Nationale, Québec, QC, Canada; 2 School of Rehabilitation Sciences, Faculty of Medicine, Université Laval, Québec, QC, Canada; 3 Réseau Autonomie Santé (RAS), Victoriaville, Québec, QC, Canada

**Keywords:** disability, diversity, equity, inclusion, physical activity, quality of participation, ultra-trail

## Abstract

*Diagonale des fous inclusive* (DDFI) was an initiative to raise awareness towards disability inclusion, generate opportunities in inclusive outdoor leisure-time physical activity, and enhance quality of participation (QoP). QoP is defined as a broad subjective evaluation of satisfaction, enjoyment, and perception of personally valued outcomes that comprises of six experiential elements, including autonomy, belongingness, challenge, engagement, mastery, and meaning. For two years, the participants in this project took part in trail events across Quebec to prepare before culminating their journey on Réunion Island with a 105-km ultra-trail, one of the biggest ultra-trail events in the world. The aim of this study was to explore QoP and the conditions that shape it among team members preparing for and participating in the DDFI. This study used a qualitative ethnographic design (descriptive and interpretative). Twenty-three participants (aged 26–67 years old; 3 people with a disability, including one adapted all-terrain wheelchair-user) were recruited using purposive sampling. QoP was explored using semi-structured interviews (*n* = 19 before DDFI, *n* = 19 after DDFI). Participant observations were conducted during preparation activities and during the DDFI. Field notes were collected during participant observations to contextualize the data. Thematic analysis was instigated using a mixed approach (deductive and inductive). Four overarching themes were extracted from data: (1) A Human Experience illustrates the life of having a diverse and inclusive group; (2) Long Way to the Top represents the reality of long-term engagement; (3) Call of the Wild encompasses a spectrum of motivations and emotions related to adhesion; and (4) Paving the Way highlights the meaning of DDFI in relation to awareness. This study offers insight into the team's QoP through the experiential elements of autonomy, belongingness, challenge, engagement, mastery, and meaning. Some conditions to the six elements were highlighted, such as inclusion, experiencing a role, group environment, and spreading awareness towards disability inclusion. Further studies are needed using community-accessible trail opportunities that are inclusive to PWDs to expand on these outcomes.

## 1 Introduction

According to the 2017–2022 Canadian Survey on Disability, 8M (27%) Canadians aged 15 years and older have a disability that restricts participation (1). Canada's 2022 Disability Inclusion Action Plan promises a modern approach to disability, with objectives to improve social inclusion and to foster a culture of disability inclusion (2). Disability inclusion extends beyond simple physical integration of persons with disabilities (PWD), such that social environments address the diverse needs through intentional efforts to ensure the success of every person (3).

The WHO Global Action Plan on Disability recognizes leisure-time physical activity (LTPA) as a significant avenue for promoting the quality of life, well-being, and social participation of PWD (4). Given a predisposition to secondary health conditions and psychosocial sequelae, the physical and psychosocial benefits of LTPA are amplified for PWD (5). However, PWD experience restricted participation in meaningful LTPA due to barriers at many levels (intrapersonal, interpersonal, institutional, community, and policy), which can hinder the quality and quantity of LTPA (6, 7). As a result, PWD are up to 62% less physically active than the general population (6). Therefore, inclusion of PWD in LTPA requires a shift in focus from physical integration to an approach that truly promotes equal access.

Furthermore, there is a stigma around some LTPAs, such as outdoor or extreme sports (i.e., ultra-trail), which commonly generate exaggerated reactions and are considered impossible or super-human (8, 9). Canadian Institutes of Health Research defines ableism as “thoughts, beliefs, actions, and practices that discriminate against persons with disabilities, based on the assumption that they are less worthy of respect and consideration, less able to contribute and participate, or of less inherent value than others” (10). This ableist discourse in outdoor LTPA could impede participation, as ableism can act as a regulatory mechanism in the participation of PWDs in sports and LTPA (11). Therefore, moving away from the ableism discourse to support empowerment is considered a priority in a call for action to support PWDs to be physically active (12, 13). One way to do so is through using a sanctioned event to provide media attention and enhance awareness about disability inclusion (14).

*Réseau Autonomie Santé* (RAS), a non-profit organization, fosters equal access for PWDs through inclusive LTPA actions (e.g, promotion, organization, coordination, and adaptation). RAS contacted the research team to evaluate the quality of their inclusive program called *Diagonale des fous inclusive* (DDFI), translated freely as *inclusive diagonal of fools*. For DDFI, RAS gathered a group of people with and without disabilities around a 30-year-old man living with the Louis-Bar syndrome, a rare neurodegenerative disorder. Together, this group experienced a shared adventure by preparing for and completing a 105-kilometer ultra-trail on the mountainous paths of Reunion Island in 2024. With DDFI, RAS aimed to set a precedent in an inclusive ultra-trail, push the boundaries of what was considered possible for PWDs and increase awareness towards the reality of PWDs. Conjointly to expanding opportunities for PWDs through disability awareness in ultra-trail, RAS wanted this expansion to be centered around the quality of opportunities, not solely on the quantity.

In the context of LTPA, Self-Determination Theory (SDT) serves as a robust framework for understanding the “why” behind participation and how the social environment shapes the quality of that experience. SDT posits that participation is not just about “how much” someone does something (quantity), but “why” they do it (quality). Conceptually, intrinsic motivation is satisfied by three basic psychological needs (autonomy, competence, and relatedness) (15). SDT is commonly applied to understand participation in LTPA, including for PWDs, notably for long-term change in LTPA behavioral outcomes (15, 16). The Quality Parasport Participation Framework (QPPF) was developed to better understand “why” people participate in parasport (and LTPA) by synthesizing literature on the experiential aspects of sport for PWDs, with its core elements heavily influenced by Self-Determination Theory (SDT) (17, 18). QPPF highlighted 6 experiential elements that should considered when assessing the participation construct: autonomy, belongingness, challenge, engagement, mastery, and meaning (17). Quality experience is the result of the appraisal of whether their participation satisfied their values and need through these experiential elements. More broadly, the quality of participation (QoP) is defined as a broad subjective evaluation that the experiences of participation, notably in LTPA, generates satisfaction, enjoyment, and perception of personally valued outcomes (17, 18). In QPPF, conditions are 25 aspects across three domains (i.e., the sport activities, the physical environment, and the social environment) that support quality experience. While some authors have explored the experiential elements and conditions of outdoor programs (i.e., paddleboard and snowsports), QoP during trail activities has not yet been documented in the scientific literature (19, 20).

Little is known about the QoP in the specific context of outdoor trails like DDFI. Literature showed that trail activities with a wheelchair user provide psychosocial benefits, such as satisfaction with health, improved social relationships, and a sense of community (21). During trail activities, pairing people with and without disabilities using a wheelchair operated by hikers increased awareness towards disability inclusion for hikers, as well as providing motivation and socialization through solidarity, camaraderie and teamwork (22). This type of activity showed a transformative power on social constructs like self-representation and identity in relation to one's disability through cooperative dynamic and a common sporting challenge (23, 24). However, a gap remains on some experiential elements and conditions to leverage for fostering QoP (15). Understanding the elements and conditions that shape QoP during an inclusive outdoor trail activities through the experience of DDFI could expand on current knowledge the context of extreme sport used for disability inclusion (6).

The general aim of this study was to explore the participation experiences of a team of people with and without disabilities during an inclusive LTPA program (i.e., ultra-trail). Two specific research aims were to: (1) Describe the conditions experienced by the team members preparing for and participating in the DDFI ultra-trail event, and (2) Explore the QoP through the lived experiences of the team members preparing for and participating in the DDFI.

## 2 Methods

### 2.1 Design

A descriptive and interpretative qualitative ethnographic design guided this research. The ethnographic approach aims at describing the culture, including the behaviors, way of life and beliefs of a group as the members sees it (25). These cultural aspects (i.e., behaviors, way of life and beliefs) can therefore be defined in LTPA for PWDs through experiential aspects of participation and the conditions that shapes them. This study followed a constructivist paradigm (26). Constructivism maintains that there are multiple interpretations of reality and that research results are constructed through the contextualised interaction between the researcher and participants (27, 28). In an ethnographic design, as the researcher remains open to the setting and subject of the study by active and sustained involvement, the researcher and participants develop a close-knit relation. Therefore, the knowledge is created in a dialectical methodology as the investigation emerges progressively and has specificity to this local setting (26, 29). The research was done in collaboration with RAS. RAS were consulted about their preferred level of involvement in every step of the research process. This resulted in high involvement in conceptualization (objectives, design, and methods) and lower involvement in data analysis (peer-checking). This study was reported in accordance with the *Consolidated criteria for reporting qualitative research* (COREQ) item checklist for interviews (30). Ethical approval was obtained from the *Comité d'éthique de la recherche sectoriel en réadaptation* of the CIUSSS de la Capitale-Nationale (#2024-3043). After receiving an *Information and Consent Form* in an accessible language, participants provided written informed consent by email.

### 2.2 Setting

**Figure 1:**
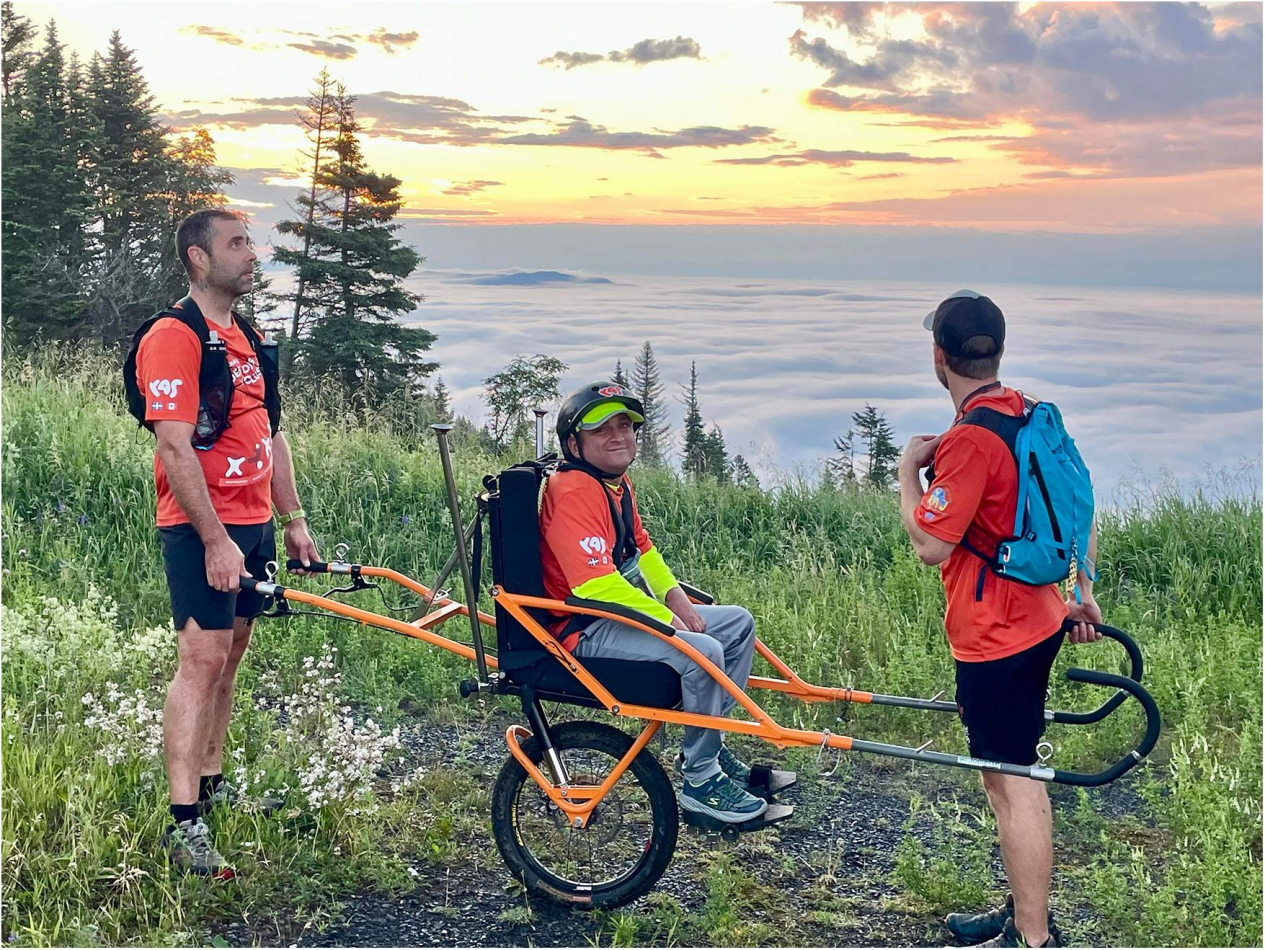
Joëlette set up, with one co-guide in the front and back of the wheelchair user.

Data were collected in Quebec City (Canada) and Reunion Island (France). Ultra-trail event *Diagonale des fous* is an international annual ultra-trail race on Reunion Island. The RAS adapted the event in 2024 to be inclusive of people with and without physical disabilities: the DDFI. This team of 31 included notably the participant living with the Louis-Bar syndrome who completed the adventure using a joëlette (i.e., an all-terrain single-wheel wheelchair, see Figure 1). This 105-kilometer 4-day event took place in October 2024 and was documented by a film crew in a documentary airing on local and national news networks. However, the DDFI program began approximately 2 years prior to the event. There were obligatory team activities and community-based LTPA for DDFI, and RAS also provided other LTPA opportunities, like wheelchair hockey or adapted rock climbing. The obligatory sessions were centered around team building and enhancing the visibility of inclusive LTPA in Quebec by participating in official and unofficial trail events. The team had two main sub-groups: the ultra-trailers, including the participant in a joëlette (PIJ) and co-guides (i.e., participants with and without disabilities who supported the joëlette during DDFI), and the members of the logistic team (MLT) who supported the ultra-trailers during the event. RAS's organisation committee was constituted mainly of 3 participants who contributed to the coordination and structure of the group. They gave participants the possibility to contribute to committees for funding (e.g., organize donation events and create a sponsor plan), physical and mental preparation (e.g., develop team building and organizing events with increasing difficulty), and communication (e.g., social media management). The team recruited two medical doctors, one of whom was attributed the role of “medical supervisor”.

### 2.3 Research team

The research team consisted of academics and representatives from LTPA organizations. RO, who is responsible for most of the fieldworks, is a man in his late-twenties, a kinesiologist (M.Sc.), and a doctoral student in rehabilitation sciences. RO initially occupied an outsider position within the group. Having joined partway through the preparation phase, he was introduced as a group member who was simultaneously conducting a research project. Nevertheless, throughout the observations and interviews, he acknowledged that his academic status, timing of entry, competencies, and research objectives shaped his interactions with participants. Group members frequently engaged him regarding the study's methods and findings, highlighting the influence of his dual role on the dialogue. The acknowledgement of the research positionality confirms the co-constructed field between the observer, RO, and the observed, the team members. The research team provided conceptual, reflexive, analytical and intellectual contributions. It consisted of seven people with various academic backgrounds, including three with extensive backgrounds in qualitative research, leisure-time physical activity and disability, and one individual in a leadership position with RAS, with a total of three men and four women. Two members of the research team identified as PWDs. The diversity among the research team fostered critical thinking and reassessment during dialogue. By reflexively considering our biases and questioning our views on disabilities, we enhanced the credibility of the results (31).

### 2.4 Conceptual framework

The QPPF was chosen as a conceptual model because it explains and facilitates the evaluation and improvement of experiences with participation in adapted activities, in this study, inclusive ultra-trail (18). It can be applied to explore experiential aspects (autonomy, belongingness, challenge, engagement, mastery, and meaning) of lived experiences. This model also highlights 25 conditions from the sport activities (e.g., funding and cost), physical environment (e.g., travel and access), and social environment (e.g., group environment) that influence the elements of quality experiences (18). This model was aligned with theorical conceptualizations of factors that contribute to well-being in general, not exclusively to PwDs (17). Therefore, it could be applied to a broad range of inclusive LTPA, notably an ultra-trail done with a team composed of individuals with diverse capacities.

### 2.5 Participants and recruitment

A purposive sample of 31 people (16–67 years of age) who voluntarily registered for the DDFI were invited to participate in the study by RO during a group meeting. He presented the research project and provided contact information (email and phone number) for those interested to follow through. In line with the concept of data sufficiency, the sample size was considered sufficient to meet the exploratory nature of the study and the study design as the whole group was approached to explore the lived experiences contextualized to this specific team (32, 33).

### 2.6 Data collection

#### 2.6.1 Participant observations

Participant observations (PO) began in January 2024 by RO. PO were conducted during all preparation activities and during the DDFI. Participants were informed of the purpose of his presence and the research interests during the first meeting. The observations were requested by RAS, which included inviting RO in the field. PO were conducted at 18 different times, analysed with field notes which were compiled in a logbook. The observer participated in all types of team activities during throughout the preparation phase, such as logistical meetings (online, about the structure and DDFI's logistics), team training sessions (in person, including team building exercises), trail races (in person, during official trail events), and committee meetings (in person, centered around physical and mental preparation). The notes were descriptive (event reports; inductive) and theoretical (analytical, deductive, based on the QPPF), organizational (understanding how the structure can affect QoP), and process-driven (focused on mechanisms rather than interactions between participants) (34). Personal notes on the emotions, impressions, or interpretations during PO were also taken separately and privately to keep records of potential biases (35). Considering the ethical issues related to observation, the observer's position followed the recommendations of Angrosino and Rosenberg (36) to connect with people who are marginalized, to question their experiences, and to become a spokesperson for their interests (37). Therefore, in a careful, open, empathetic, and collaborative posture, the observer frequently questioned the PWDs about their experience in DDFI and life to connect with them.

#### 2.6.2 Semi-structured interviews

Semi-structured interviews were used to explore the individual experiences and conditions related to the DDFI through the perspective of the team. The interviews of approximately 60 min were conducted during preparation activities (*n* = 19) and after the DDFI (*n* = 19). The “before” interviews took place approximately 6 months after the start of the participant observations, and 3 months before the DDFI. The participants were therefore familiar with the interviewer. The “after” interviews took place approximately 3 months after the DDFI to allow time for reflection on their experience. Participants were given a choice of interviewers between RO (male, mid-twenties, participant) or JB (female, mid-twenties, non-participant) to limit the possible challenges posed by the presence of RO as a male participant and to provide a choice (31). Interviewers were trained by experts through a graduate level university course in qualitative methods and through individual and customized sessions with an expert who runs a qualitative research platform. The interview guides were pilot tested by students (*n* = 2) who were aware (1) or participated (1) in an activity of the DDFI program. One-on-one interviews were favoured. The semi-structured interview guide (in Supplementary Material) was developed by RO, JB, and JB. It contained 9 primary open questions with 3–5 elicitation questions for every primary question. It was structured around the QPPF, exploring experiential elements of QPPF (e.g., How do you perceive your autonomy and level of involvement in preparing for the DDFI? How does preparing for the DDFI challenge you?) (18). Interviewers remained flexible to unexpected and emergent topic related to the research questions. The interview guide before the DDFI was centered on the preparation activities (up until travel to Reunion Island), while the post-interview was centered on the Reunion Island trip, including travel from Quebec and the DDFI event. Interviews were conducted in person or online (Microsoft Teams, 2025), depending on participants' preference, and were audio-recorded.

### 2.7 Data analysis

A reflexive thematic analysis was conducted following the six phases described by Braun and Clarke (38, 39). Interviews were transcribed from the audio records by 3 different members of the research team (RO = 24; JS = 10; JB = 4), who then familiarized themselves with the data by relistening to audio file and re-reading the transcripts. Transcripts were coded line-by-line using NVivo software by RO (40). A mixed approach was used by RO, where a codebook was developed deductively around the 6 experiential elements and 25 conditions of QPPF, with additional codes and sub-codes added through inductive inquiry. Two members of the research team (RO and JS) coded ten transcripts and discussed the codebook and meanings to enhance confirmability (31). Although QPPF was initially used to deductively code each transcript, the members of the research team remained cautious to keep the extracted content in context to enhance credibility (31). The personal notes on the emotions, impressions, or biases taken during PO were reconsulted before generation of themes. Some elements of positionality around RO (e.g., the influence of being a physically active male without a disability on the team or the development of a varying friendship with different participants on his interpretation) were discussed with the research team. Then, RO explored the codebook reflexively to extract overarching themes with shared meaning for internal homogeneity (38, 39). Considerations that emerged from the discussions with the research team were deliberated during the initiation of themes for credibility, such as: the fair representation of every participants' point of view, the right representation of PWDs in themes, and the impact of the active involvement with RAS in the research (31). A convergent method triangulation with the logbook was used to increase authenticity of knowledge and to generate initial themes (37, 41). Observation notes were extracted to contextualize the strategies implemented in connection with the interviews. Peer checking with the research team and member checking of final themes and sub-themes (6 of the interviewed participants) were performed to enhance trustworthiness (42). In this way, we ensured the co-creation of knowledge by allowing participants to engage with our interpretations of their data (43). Themes were constantly compared with their meaning, elements, and conditions to ensure external heterogeneity (38). The themes and sub-themes were reviewed iteratively until a final selection was made (42). The overarching experiential elements were extracted from the final themes. In order to preserve the anonymity of participants and considering the potential risk of identification based on their characteristics, solely the participants' subgroups (i.e., co-guides and MLT) were reported. PWDs were consulted to ensure they agreed with their statements.

## 3 Results

The final sample consisted of 23 participants aged 26–67, including 20 ultra-trailers, the PIJ (PWD) and 19 co-guides (1 PWD), and 3 MLTs (1 PWD) who participated in a minimum of one interview. Thirty-five (35) interviews were conducted using Microsoft Teams, and 3 were conducted in person. One co-guide completed both interviews (before and after DDFI) despite leaving the program during the DDFI for personal reasons. One participant refused to participate in the project due to time constraints, and other participants (6) did not contact the research team. One interview after the DDFI was conducted as a pair at the participant's demand.

Four overarching themes emerged from the analysis: (1) A human experience, (2) Long way to the summit, (3) Call of the wild, and (4) Paving the way. Table 1 presents the description and experiential elements of each theme. The organization of themes consists of an overarching concept containing experiential elements and conditions during preparation and DDFI unified by meaning. As the QPPF highlights the variation over time of the value placed on each quality experience element, the sub-themes (preparation and DDFI) highlighted specificities during each moment of the interviews. Table 2 presents the identification of conditions for QoP from data analysis. Examples for each identified condition are found in the Supplementary Material.

**Table 1:** Characteristics of the themes.

Themes	A Human Experience	Long Way to the Summit	Call of the Wild	Paving the Way
Description	This theme highlights the benefits and challenges imposed by having a diverse and inclusive group.	This theme highlights the conditions for long-term engagement, both individually and group related.	This theme highlights the effect of a challenging adventure and being in nature on the flow and mastery.	This theme highlights the meaning that participants give to the DDFI related to mentorship and awareness.
Experiential elements	Belongingness	Autonomy	Challenge	Autonomy
	Challenge	Belongingness	Engagement	Meaning
	Mastery	Engagement	Mastery	
		Mastery	Meaning	

**Table 2:** Conditions to quality of participation from QPPF that were identified as a code in the interview code tree or in the logbook from participant observations.

Conditions to QoP	Code tree	Logbook
Physical environment		
Accessibility (physical and services)	●	●
Travel and Access	●	●
Safe Places	●	●
Access to Equipment	●	●
Within the activity		
Sport type	●	●
Program size	●	●
Funding and cost	●	●
Options	●	●
Individual level of challenge	●	●
Safe Activities	●	●
Classification	●	
Inclusiveness and similarity	●	●
Social environment		
Coach's knowledge, skill, and learning	●	●
Autonomy support	●	●
Tracking athletes improvement	●	●
Develops role	●	●
Interpersonal skills of coach	●	●
Group environment	●	●
Mentorship	●	●
Familial support	●	●
Educating parents and family members		
Harassment	●	●
Sport-related attitudes	●	●
Status of parasport	●	●
Unique pathways*		

*This condition does not apply to Diag because it is a one-time program, not a recurrent one.

### 3.1 Theme 1: a human experience

A human experience primarily explained the experiential elements of belongingness, challenge, and mastery. As a co-guide with a disability explained, this theme highlighted how the inclusion of diverse people, young and old, with different capacities and from diverse backgrounds, creates a safe space for the *mastery* of personal competencies:

What will come out of this is a lot of human experience. The pretext is a physical challenge, and it is really important. But it's all about inclusion, which is exemplified by our entire team, and how things unfold, how we interact with each other, and how we complement each other. I find the human experience so enriching.

The inclusion of all profiles allowed them to learn from people they would not normally have talked to and to change their outlook on life, both socially and personally. However, participants noted that this diversity meant working with a variety of personalities, which led to interpersonal conflicts that could negatively impact individuals and the group dynamic. As a co-guide said:

We're such a big group, and a diverse one at that. Like I said, I think all of this is bound to cause some friction. It can't be smooth sailing, and that's totally normal. […] Then again, in any team, there will always be conflicts, differences of opinion, differences of mentality.

Participants mentioned certain conditions that influenced unity, such as humility, communication skills, sensitivity to others, setting goals and values as a team, and sincerely embodying them at critical moments. One co-guide expressed:

What is most important for a quality experience is defining clear goals and clear values as a team and then maintaining fluid communication throughout.

#### 3.1.1 Preparation

In the preparatory moments leading up to the DDFI, having a variety of activities and opportunities for team building, training, and consolidation was an important factor influencing group cohesion. These group moments created foundational memories that were key to *belongingness*. In the logbook, examples of activities were logistical meetings, trail running across Quebec (e.g., Mont-Mégantic), team-building activities (e.g., a 24 h sleepless weekend), fundraising activities (e.g., cross-training and auction event), and having support for team building (e.g., hiring coaches with expertise in group cohesion). A co-guide explained:

In building relationships with others, I believe that during team moments, team dinners, team activities, in short, having that connection with others. […] You really get the impression that deep down you're sharing something.

Stability of group composition was important to ensure a healthy group environment and allowed for deeper relationships within the group. In the logbook, the authors noted that three participants left the group during the participants' observations, including one during the DDFI at Reunion Island. Certain interpersonal skills were brought up as critical for *belongingness*, such as communication, adaptability, open-mindedness, and consideration of the group in the decision-making process. One co-guide with a disability described how these departures affected the experience:

Actually, it's been pretty tumultuous for me. In fact, there were a lot of changes [of team composition] in the first year. For the last six months, it's been much more stable, and I think that's good.

#### 3.1.2 DDFI

During the DDFI, the human experience was described as the result of sharing with inspiring people who pushed themselves in important moments to create lasting memories. The participants highlighted how a group that helped each other, engaged in caring for people with special needs, and cooperated in difficult times (e.g., illness or great fatigue) restored faith and developed *mastery*. For example, a co-guide noted:

I saw sacrifice, I saw compassion, I saw tolerance, respect, mutual aid, and solidarity. […] Ultimately, all this well-being that you see, it enriches me, it makes me feel good.

The strategy of assigning two-person teams (i.e., someone to take care of each other) was also seen as helpful to the experience:

The physical training team formed pairs. I thought about it again recently, and how it was a plus […] we take better care of others than we do of ourselves.

Certain pivotal moments affected the meaning of this experience, such as a section of the trail completed without PIJ (PIJ had to take a car for a portion of the trail because of its inaccessibility despite using a joëlette) and mourning the end of the adventure with the group.

I think there is a stage of grief that I hadn't really anticipated. It is difficult to let go of these wonderful people … because the reality is that we are in the midst of our lives, our families, in our little corners of the world…

### 3.2 Theme 2: long way to the summit

Long way to the summit highlights the interrelatedness of *autonomy*, *challenge*, *engagement*, and mastery during DDFI. The DDFI was an experience that took place over up to two years for some participants. The *engagement* needed to carry out such exhausting activities over such a long period of time required strong motivation, goal setting, and a balance with personal life. When asked about which aspects of the DDFI were *challenging*, one co-guide answered:

The patience to undertake such a long-term project. […] There is a difference in how I manage my own personal life, and when I am in a collective group. […] Sometimes, I get a little impatient because I envision things moving a little faster.

Factors that participants raised as important for the team included the structure and logistics, experiencing a valued role within the team, and having mechanisms in terms of leadership, committees, medical support, challenges, and communication methods. When asked about his perception of *autonomy* over his sport involvement and contribution within the team, one co-guide expressed:

It's one brick in a brick wall. Everyone contributed. Everyone had a role to play.

Some participants highlighted the need to have an organic and flexible process despite providing structure, to navigate the unknown that was inherent in the complexity of the activity. A co-guide with a disability mentioned the need for structure and leadership vision to be adapted to the profile of the participants.

In some situations, you need directive leaders[…] For me [Participant], he is a unifying leader, you know. […] That's the type of leader that appeals to me more.

#### 3.2.1 Preparation

Participants had a schedule that involved bi-weekly activities in 2024 and had to plan the logistics of the DDFI together. The group environment and interpersonal skills were cited as notable social conditions. A co-guide described:

What's most important when you are with a group like that… It's getting together. […] It's all about being a group.

In addition, experiencing a role that was valued both personally and within the group was a condition to *engagement* during preparation. Group management, using adequately the complementary skills brought by the diversity, participant involvement in committees, and support for *autonomy* were mentioned as conditions that impacted the *engagement* in this type of initiative. In the logbook, it was noted that funding, physical preparation, and mental preparation were major points in the schedule during the logistics meeting with allocated time to discuss initiatives from sub-committees (i.e., sharing resources and a financing strategy). This *engagement* allowed the *mastery* of key skills, such as specific carrying techniques, group management, disability-related skills, and mutual trust as described by the PIJ:

As we practice more, it becomes easier to ask for things based on my needs, based on the joëlette, or based on the group.

Some participants pointed out that talking to a group with experience in inclusive trail helped them feel more secure in their personal planning and team expectations. In addition, having the right information about the *challenge*, establishing routes that facilitated progression in the level of physical *challenge*, and representing *meaningful* goals were considered important for quantifying progress, *mastery,* and motivation. This progression was highlighted by a co-guide with a disability:

You know, I knew that my biggest challenge was going to be … my perception of not being able to contribute physically. Knowing that before [the accident], that was one of my strengths, […]I mean, a big challenge like that … I'm climbing big steps … There's a lot of climbing on the trails … but I also have a lot of climbing within myself, in my process.

#### 3.2.2 DDFI

Slowly, then all at once, two years of *engagement* came to fruition over ten days on Reunion Island, including approximately 66 h of *challenges*. A co-guide explained how the exhaustive preparation allowed the group to have the right level of *challenge*.

I thought to myself, two years of preparation for four days. I found it a bit ridiculous; I thought it was far too long. But in the end, that preparation really paid off during the event … because we felt supported. We really felt like a great, close team … a big family, in a way.

Individual weaknesses and difficulties were overcome by the collective drive, cooperation, and mutual support. A non-judgmental environment created by those more involved in the carrying was considered important to allow for inclusion and diversity despite a physically draining task. In return, being able to pitch in after going through more difficult times marked the experience of the co-guides.

We could also roll more [rolling the joëlette means using the wheel instead of carrying it off the ground], carry less … the fact that I could roll made me feel strong, and then I managed to recover. That's one of my … best moments. It's one of my highlights to be able to get back on the joëlette and say, “I can do this.”

### 3.3 Theme 3: call of the wild

Call of the Wild encompassed the elements of *challenge*, *engagement*, *mastery*, and *meaning*. The participants valued having a personal goal in the social adventure of the DDFI. The *Diagonale des fous*, considered the “*Holy Grail*” by one co-guide, and the appeal of a unique trail running adventure, were things that resonated with the participants' own physical or spiritual goals. Talking about the reasons that motivated her *engagement*, a co-guide highlighted:

The fact of initiating a movement [in inclusive LTPA] it's already better than being in neutral. That's why I'm involved, but of course, there's also the notion of adventure. If you want to advance a cause, I think it has to be through something that inspires you [referencing ultra-trail], and that's going to motivate you.

For some, like this wheelchair user and MLT, these goals were related to their professional lives:

My personal goal was really to develop inclusive sports, because that's what my job is about, because it's part of my life mission.

For others, the project was linked to their sporting or personal journeys. For example, one participant emphasized the important role his deceased son, who had a disability, played in his *engagement* in DDFI.

He's no longer here today. You know, I've been through something. It [DDFI] makes you start digging into your regrets, your remorse, and it helps you rebuild.

The concept of living in the moment and the importance of seizing opportunities that arose, referred to as the “*urgency of living*” by one co-guide, was a pillar in this theme. This was a lesson for co-guides, which was intrinsically linked to their cooperation with PWDs. The PIJ expressed the importance of living life to the fullest and that a group was essential to fulfillment in his life:

When you have the opportunity to go out and see the world, you have to go for it […] I'm not supposed to have been here for 20 years [making reference to his disability, which predicted a shorter lifespan], so I enjoy everything that comes my way. I have legs, but they [the members of the team] do what I can't do. They walk where I can't, they take me to places I wouldn't have gone if it weren't for the group.

#### 3.3.1 Preparation

Taking on a *challenge* in a supportive group environment, surrounded by nature, was a condition to feeling *engaged* (i.e., staying focused and living in the moment). Some participants noted that time passed more slowly when the group was too focused on performance, during interpersonal conflicts, and during moments of group indecision. When talking about the interactions with other members of the team, one co-guide raised:

It was the little things that accumulated. Why did I have to deal with them? […] These various issues [conflict situations] that arose prevented me from enjoying the moment.

On the other hand, some co-guides described how close contact with nature and deep conversations with others allowed the immersion in the activity and gave the impression that time was passing quickly.

Yeah, I think it’s the setting [in the outdoors], the group, the activity that makes it easier to be in the moment and appreciate everything that's happening there.

#### 3.3.2 DDFI

The DDFI allowed participants to experience a range of positive and negative emotions, including joy, pride, anger, excitement, and discouragement. One co-guide described the spectrum of the emotion felt during DDFI:

We went through quite a range of emotions, you know, from total happiness to total anger. I don't have the whole spectrum of emotions here, anxiety, stress. And as I said, joy, happiness, elation, then distress, discouragement.

It allowed the participants to live an experience aligned with their values, which resulted in cries of joy and sadness, and to realize how lucky they were.

What stands out most is joy. A joy that I have probably never felt in my life. Okay, I'm going to be emotional again, but you know, going on a trip is already powerful, doing a trail is powerful, doing all of that inclusively with a group is exponential.

However, according to participants, it was the moments of great adversity, including conflicts and hardships, that generated the most pride. Such that facing a major *challenge* led to a more grandiose success, as explained by a co-guide:

What's beautiful about our story, and about our experience, is that sometimes things went wrong or we were worried. But after that, the emotion of success is even more spectacular than if everything had gone smoothly and we hadn't had any doubts.

### 3.4 Theme 4: paving the way

Paving the Way incorporates the elements of *autonomy*, *belongingness*, and *meaning*. The participants saw the DDFI as a way to set a precedent, to be used as an example to promote sport for all, and to influence how people perceive inclusion. To set a precedent, flexibility and openness were required on several levels to overcome medical and insurance barriers, as highlighted by a MLT:

He chose to live. We chose to support him in what he wanted to experience. But first, we had to fight with the doctors because we needed a medical report stating that he was fit to travel. The doctors were very hesitant…

The visibility was important to some participants because the DDFI included a few PWDs and is financially and logistically complex to replicate in its entirety.

It benefits one person. You know, what could we have done [for inclusion of PWDs] with [Cost of the initiative]? We were betting on visibility; that's how you have to look at it.

DDFI also required flexibility and adaptation in the rules of the activity, which could influence the difficulty for PWDs, as explained by a co-guide with a disability:

They also need to be flexible in terms of rules, like with walking sticks [members of the team were allowed to have them despite being officially prohibited during the event], to allow people to complete it and be inclusive.

#### 3.4.1 Preparation

Preparing for the DDFI sometimes involved running on trails during organized races and sometimes running freely. Interacting with other runners at events and the perception of the community were a *meaningful* part of the experience for participants as described by a co-guide:

You know how many people kicked us in the butt [when the group was on the trails], you know, a little angrily […] then when they got to PIJ, the tone changed completely, “Wow, congratulations, don't give up, it’s cool,” you know, seeing these people […] I think it's also great that we opened the eyes of organizations to this inclusion.

In order to amplify the impact of DDFI, participants emphasized the importance of spreading the message beyond the challenge itself. Activities carried out in public, radio interviews, television appearances, a visit to Parliament with their depute, the “research project”, the documentary, and the film, all of which are noted spin-offs of the project because of the connectivity of team members, extended the perceived impact of the project for the cause.

Being in the media, having a platform to talk about it, you know, with reports, “Salut, Bonjour”[a television show], the documentary that’s going to be released, and I really feel that having a nice emphasis, a nice visibility on this cause, it's going to promote all the other activities that are going to come, you know.

#### 3.4.2 DDFI

The relationship that participants developed with (PIJ) was a cited source of motivation and a lever for learning. Being able to cross the finish line and complete the adventure as a team, but above all with PIJ, opened the eyes of co-guides to the plethora of possibilities when people put their minds to it.

Seeing [PIJ] cross the finish line, at that moment, I told myself that nothing is impossible in life.

Participants emphasized that the DDFI should not be seen as an end goal, but rather as a step along the way to inclusive outdoor leisure. They raised the importance of continuing to get involved, volunteering, and developing inclusive outdoor LTPA through various activities in their community. For example, a co-guide explained her willingness to pursue trail running using a joëlette:

In our community, I want to see this [trail running using a joëlette] develop, and I'm going to do everything I can. I'm going to do what I can, and I'm going to put my contacts to work.

## 4 Discussion

RAS used the DDFI as a means to set a precedent for inclusion in extreme adventures and by increasing awareness about the reality of PWDs in outdoor leisure. They gathered an intergenerational group, with and without disability, around a 30-year-old with a neurodegenerative syndrome, with the goal of completing the DDFI around a group culture of inclusion. This study explored the lived experiences, the QoP and conditions to QoP, of the team members preparing for and participating in DDFI. The novel contributions of this study lie within the uniqueness of the activity, the diversity of the sample, and the design.

The QPPF highlighted the interrelatedness of the quality experience elements (18). Although asked as separate constructs in the interview guide, these elements in DDFI were strongly interrelated to form a coherent yet complex product unified by meaning. The participants described the quality of their participation with a holistic view around certain central concepts. An example is the theme Call of the Wild, where having the right amount of challenge and belongingness was connected to engagement. This was also the case during a dance therapy program for PWD, where the participants connected the concept of engagement as the product of challenge and mastery (44). In this way, the four themes contained most experiential elements, with an accent on some of the elements for every theme (18). The experiential elements guided the analysis and explained the quality of their participation for both PWDs and participants without disabilities. It showcased the pertinence of the QPPF in a broader inclusive concept, with nuances in the expression of the elements compared to disability-specific programs seen in the literature. Adapted LTPA has a range of possibilities to consider in terms of inclusive experience, from adapted leisure in specialized areas to inclusive leisure in the community (45). Specialized community programs for adapted LTPA in the literature highlighted the importance of relatedness for belongingness when programs offer a moment to connect with people in similar capacities (20, 46, 47). The participants in the DDFI were different in age, capacities, and fitness. The inherent challenge of the task imposed a high number of co-guides to accomplish the goal, therefore operating in an inclusive context. While DDFI lacked similarity for PwDs, the diversity brought belongingness that was key to mastery. This diversity also brought variety in the importance of each element to QoP. The QPPF also highlights the varied value of experiential elements (i.e., the value placed on experiential elements may vary from person to person) and the varied means of achievement (i.e., individuals may seek out different strategies to achieve a quality experience) (18). Therefore, the subjectivity of quality participation must be considered when interpreting the results of the manuscript, where the overarching themes resonated with participants to varying degrees (18).

In DDFI, the varied value of experiential elements and varied means of achievement also applied to conditions (18). An overwhelmingly large proportion of the expressed conditions were considered as facilitators or barriers to QoP by different participants and therefore described in the neutral form in the results. For example, the required structure (i.e., group rules) and leadership for the completion of DDFI varied greatly, from being considered a barrier to a facilitator to their experience. For others, the need for structure was considered less important for experiencing quality participation. Ultimately, as a co-guide with a disability mentioned, what's important is that the conditions to QoP are adapted to tailor the needs of the participants, to what they consider appealing. The conditions to QoP that were identified by the participants affirmed the ones expressed in QPPF and introduced novelties (18). Similarly to other team programs, providing social opportunities outside of the sport and providing opportunities for feedback conditioned belongingness and autonomy (48). Like other outdoor adapted sports such as paddleboarding, factors such as funding and program aversion to risk were important in the DDFI (49). The unpredictable nature of being outdoors, especially when engaging in extreme activities that involve a certain amount of risk and uncertainty, is intricate to the structure and implementation of initiatives such as DDFI (50). This was observed with the PIJ's difficulty in getting his doctor's approval, which was required for insurance. Additionally, as discussed previously in a veteran LTPA program and trail activities, the group environment, cohesion between members, and the empowerment through contributing to a valued role were notable conditions for long-term engagement and feeling a human connection for DDFI (22, 51). Some conditions were novel and specific to the motivation of using the DDFI as an awareness event for visibility and inclusive sport status. The perceived impact through local news, the film crew, and local political deputies enhanced the meaning for participants. Therefore, promoting visibility and awareness of the inclusion of PWDs through an inclusive LTPA program could contribute towards a better QoP in such program. These conditions resonated with the Parasport-leveraging Framework, which aims at leveraging small-medium scale parasport events for social participation and community outcomes (52). This framework could also provide guidance on these conditions for the uptake and sustainability of a similar program in the future.

DDFI highlighted the usefulness of the QPPF in an inclusive team around a wheelchair user who completed a 105-kilometer ultra-trail on the mountainous paths of Reunion Island in 2024. However, the subjectivity of QoP, interrelatedness, varied value of experiential elements, and the varied means of achievement were key considerations in the analysis. The conceptual framework is inherently complex as it defines QoP as a broad subjective evaluation of satisfaction, enjoyment, and perception of personally valued outcomes (18). Within an inclusive program, the diversity of profiles brings a diversity in what is considered important to QoP. Nonetheless, the participants of DDFI had their experience influenced by experiential elements and conditions to QoP. In DDFI, the elements were fostered through human connections, overcoming a long summit, a challenging and motivating adventure, or a personally and socially meaningful experience. In addition to creating awareness, the results may inform future practices about the inclusion of people with all types of ability in extreme outdoor events. We recommend future research to explore elements and conditions of QoP that could be enhanced to improve program delivery. Moving from evaluation to application, we recommend the implementation of strategies that target specific QoP elements and conditions for initiatives like DDFI (53, 54). Finally, DDFI exemplified the benefits of inclusion and diversity of all profiles within an extreme outdoor sport. On a societal level, initiatives such as DDFI could encourage a shift away from ableism discourse, further facilitating participation of PwD in such extreme activities. Doing so could contribute to the realization of disability inclusion, making LTPA accessible in a variety of opportunities.

### 4.1 Limitations

Although this study presented advancements in knowledge concerning QoP and its conditions during ultra-trail events and disability inclusion, some limitations must be considered. By the active involvement of the author (RO) within the team, the study design, and the constructivist research paradigm, the study outcomes are group and context-specific. They are the results of the interaction between all involved members, which limits the transferability of outcomes (31). Moreover, participants were a heterogeneous group (i.e., for age and capacities), limiting the applicability for a specific population. The sample size in this study included a small number of participants with a disability, which limits transferability. Additionally, the study used the QPPF that was designed specifically for PWDs and applied it to people with and without disabilities, which may decontextualize certain data in order to fit them within a framework despite further inductive inquiry. Finally, DDFI's organisation committee members were considered as participants, and one of which was also a member of the research team (ES). However, this shared leadership is key in participatory research, in which the research process is done with people/communities rather than on them (55).

## 5 Conclusion

This study explored the participation experiences of a team of people with and without disabilities during an inclusive LTPA program (i.e., ultra-trail). The team member's QoP was described through the experiential elements of QPPF, particularly through belongingness, meaning, mastery, and engagement. Some conditions to the six QPPF elements were highlighted, such as inclusion, experiencing a role, group environment, and spreading awareness towards disability inclusion. The subjectivity of QoP, interrelatedness, and varied value of experiential elements, and the varied means of achievement were key considerations for quality participation. Further studies are needed using community-accessible trail opportunities that are inclusive to PWDs to expand on these outcomes.

## Data Availability

The datasets presented in this article are not readily available because the Confidentiality, Storage, and Use of Results section of the Ethics Committee prohibits the disclosure of written data beyond the scientific publication. Requests to access the datasets should be directed to krista.best@fmed.ulaval.ca.
