# Supplementary material for: Exploration of the quality of participation in an inclusive ultra-trail initiative for people with a disability: a qualitative ethnographic study
Source: Front Sports Act Living. 2026 Jun 5;8:1771945. doi: 10.3389/fspor.2026.1771945 (PMC13279626; doi:10.3389/fspor.2026.1771945)
Supplement: Supplementary file 1 [file Table1.docx]

| Conditions to QoP | Description in the Canadian Disability Participation Project (CDPP) blueprint^1^ | Examples of codes from the interview code tree | Examples in the logbook from participant observations |
| --- | --- | --- | --- |
| Physical environment |  |  |  |
| Accessibility (physical and services) | Facilities are set up to allow access for participants. | The accessibility is compensated for by the group | There were non-accessible environments during many events (i.e., stairs only during the 1^st^ observation and 2^nd^ observation, high angle in the slope of the terrain in Reunion Island). |
| Travel and Access | Sport setting is conveniently located for participants; participants have ease of access to the sport setting. | Frequency and diversity of meetings;  Geographical proximity of the activities. | It was noted that some activities lasted up to 24h and took the whole weekend, while others started early (from 1 am to 3 am).  In the 48 days between May 20^th,^ 2024, and July 7^th,^ 2024, there were 7 organized activities, highlighting a high solicitation. |
| Safe Places | Participants are assured that the facilities and location are safe. | Insurance and medical approbation | The participants signed moral contracts before participating in which there was a statement of responsibility for potential injuries or damages. |
| Access to Equipment | Participants have access to equipment that suits their needs or competitive level. | Flexibility in the rules | RO and a participant were authorised to use walking sticks because of a condition that affected mobility. |
| Within the activity |  |  |  |
| Sport type | The activity is valued by the participants | Being in nature;  Connection to one's life. | During the debrief done after the trail activities, some participants mentioned positive elements of the activity. |
| Program size | The program is large enough to have self-sustaining membership and provide varying ways to be involved | The mission requires a team;  Opportunity, or not, to have a role in the team. | Some participants had a pre-determined role in committees (i.e., funding, mental and physical preparation) beyond being a co-guide when RO began participant observations. |
| Funding and cost | Keeping the cost as low as possible, funding to support involvement | Funding and cost. | The funding committee was established as a way to offer opportunities to reduce the cost burden of participation (i.e., with an investment plan with benefits)  There is a monetary commitment to the participation of $1,000 per participant. |
| Options | Participants have the choice among different sports at varying skill levels | Opportunities for trial and error. | The team took the opportunity to try different carrying methods during the Quebec Mega Trail to overcome obstacles.  After trying a method of carrying the joëlette in pre-determined pairs, a more self-determined mode was kept for Diag, where one can modulate their turn of carrying based on their sensations. |
| Individual level of challenge | The activity is designed to appropriately challenge the participants and push out of comfort zone | Progressive preparation in intensity, frequency, and duration. | The team progressed from a one-day activity with the joëlette to consecutive days of activity during the preparation for DDFI. |
| Safe Activities | Comfortable level of risk for each participant and limit the potential for perceived physical harm risk | Injury, falls, fatigue & Safety;  Getting involved in the care of [PiJ]. | Some precautions were taken to increase the safety of the activity (i.e., PiJ was wearing a helmet and a seatbelt).  There was a fall risk for co-guides, especially in wet terrain (some falls were registered in the logbook). |
| Classification | Equal opportunity and fair competition, Must have somewhere to fit | Selection of participants with similar motivations. | Not found in the logbook. |
| Inclusiveness and similarity | Integration of able-bodied athletes or differing abilities, but provide chances for interactions with participants with same level of capacities | Participants helping each other during difficult times;  Inclusiveness and similarity. | It was noted that during team-building activities, adaptations were made and offered for participants with a disability. |
| Social environment |  |  |  |
| Coach's knowledge, skill, and learning | Technical knowledge and skill related to the sport and the specific disability context to design appropriate activity | Group management skills;  Planning while navigating the unknown; | A meeting was held with a team from France who previously did the Diagonale des fous with a joëlette. |
| Autonomy support | Find ways for athletes to perceive control over program or activity | Structure, committees, and logistical support;  Debriefing & opportunities for group feedback. | The team explicitly mentioned the role of PiJ and his responsibilities.  Changes in co-guides around joëlette were made according to their wishes instead of working with specific dyads with a “working time”. |
| Tracking athlete's improvement | Benchmarking or encourages self-monitoring so the athlete know when they improved | Encouragement and preparation;  Specific training for Diag. | During a coaching session on group environment, every participant had to choose a personal objective they wanted to improve until Diag. This personal objective was brought up in follow-up activities and Diag. |
| Develops role | Encourages individual members to take on unique roles that are vital for the group and these contributions are recognized | Dyadic pairings;  Assignment of roles (collective and freely versus imposed). | New and specific roles were assigned the day before Diag, including parts of the leadership, coordination, dyadic pairing, medical decisions, moral support, and feeding coordination. Unlike committees, these roles were assigned without consulting the team. |
| Interpersonal skills of coach | Supportive leadership style and fosters positive relationships with participants that engender trust | Communication and transparency;  Interpersonal skills; | The organizing committee of the Diag started monthly online meetings to discuss funding, calendar of activities, updates on documentations, and other. |
| Group environment | Members represent a cohesive group that feels united as they pursue shared goals | Dis.united by a clear goal and values, thinking as a group;  Group environment. | During an activity at the beginning of the year 2024, the team decided to determine the 4 core values: cooperation, respect, perseverance, and goodwill. |
| Mentorship | Opportunity to mentor others to contribute to teaching/learning process | Share your experience with inspiring people who push themselves. | Participants had opportunities to contribute to teaching through the monthly online meetings (i.e., by sharing their training program), but also in debriefs after the activities (group feedback to allow solution finding). |
| Familial support | Family members support sport involvement and provide support in an autonomy-supportive manner | Familial support | Strong involvement of PIJ’s family in the process (i.e., PIJ’s mom is in the logistics team, they offered refreshment stations during activities, went to Reunion Island, and they participated heavily during funding). |
| Educating parents and family members | Sport organization provides family members with opportunities to seek education regarding parasport | Not found in codebook. | Not found in the logbook. |
| Harassment | Sport environment is free from harassment and discrimination in all social interactions | Communication and transparency. | In a letter to all participants, one participant who left the Diag mentioned gestures that lacked consideration and respect for this person’s health issues. |
| Sport-related attitudes | Perceived genuine societal attitudes toward parasport, particularly from volunteers | Positive perception of the community and crowd. | Public awareness activities were generally well-received, and there was strong funding support. |
| Status of parasport | Experiencing equal treatment and support when participating alongside able-bodied competitions | Status of parasports;  Attitudes of runners and organizers toward adapted sports. | Special status did not allow eating at the aid stations during some races.  The organizers removed the trail markers of the official path before the teams finished completing one official race. |
| Unique pathways* | Varying sport pathways and have support to pursue their individual pathways, ranging from elite to recreational | Not found in codebook. | Not found in the logbook. |

*This condition does not apply to Diag because it is a one-time program, not a recurrent one.

1. Canadian Disability Participation Project. (2018). Blueprint for Building Quality Participation in Sport for Children, Youth, and Adults with a Disability. University of British Columbia, Kelowna, BC. Retrieved December 8, 2025, from <https://cdpp2.ca/publications/a-blueprint-for-building-quality-participation-in-sport-for-children-youth-and-adults-with-a-disability/>
