# Supplementary material for: Exploration of the quality of participation in an inclusive ultra-trail initiative for people with a disability: a qualitative ethnographic study
Source: Front Sports Act Living. 2026 Jun 5;8:1771945. doi: 10.3389/fspor.2026.1771945 (PMC13279626; doi:10.3389/fspor.2026.1771945)
Supplement: Supplementary file 2 [file Table2.docx]

# Interview guide post DDFI

We invited you to this interview today because you participated in the Diagonale des fous inclusive (DDFI). We would like to learn more about the quality of participation during DDFI, the obstacles and facilitators, and the effects of this event on the social participation of people with and without physical disabilities.

As indicated in the consent form, the audio and video of this session will be recorded and transcribed. I will let you know when I start recording. If a name appears in the interview, it will be removed from the transcript and replaced with a pseudonym. Do you have any questions before we begin?

I will now start recording.

[*If it is their first interview*]

**Introductory question** — Tell me a little about yourself.

Elicitation questions:

a. What do you do for a living? School, work, etc.

b. How do you like to spend your free time?

c. What activities do you like to do in your free time?

d. What other hobbies, sports, or recreational activities do you participate in?

e. When did you start participating in DDFI?

f. Is there anything else about yourself that you think is relevant for us to know?

[*Questions for everyone*]

**Question 1.** How did the inclusive Diagonale des Fous go?

Elicitation questions:

a. Did you consider it a positive experience?

b. How did your experience differ from the preparatory hikes?

c. What were some of the barriers you encountered during the DDFI?

d. Can you give examples of strategies you implemented to overcome these barriers?

**Question 2.** How did you perceive your autonomy and level of involvement in the DDFI?

Elicitation questions:

a. How were you involved in decisions during the DDFI?

b. What was your role in the DDFI?

c. What factors influenced your autonomy and level of involvement?

d. What strategies should be implemented during initiatives such as the DDFI?

**Question 3.** How did the DDFI influence your sense of belonging to the group?

Elicitation questions:

a. How did your relationship with the group evolve from the beginning of your involvement to the end of the project?

b. What factors affect whether or not you feel like an integral part of the group?

c. What role or importance did the “inclusive” aspect of the adventure have on the quality of your experience?

d. What are the main barriers to implementing an inclusive approach like yours?

**Question 4.** How did the DDFI challenge you?

Elicitation questions:

a. Physically?

b. Socially?

c. What are the accessibility and inclusion challenges for people with reduced mobility?

d. How did the DDFI challenge your abilities?

e. How did you perceive your skill level in relation to the level of challenge?

**Question 5.** What skills have you acquired through your experience at DDFI?

Elicitation questions:

a. How would you describe the evolution of your inclusive outdoor skills?

b. What lessons can be learned from your new skills?

c. What were the successes?

d. What skills were key to meeting the challenge?

**Question 6.** What were your goals? Do you consider that you have achieved them?

Elicitation questions:

a. How do you feel about achieving/not achieving this goal?

b. What factors influence your perception of DDFI?

c. How do you perceive the impact of DDFI

- On participating persons with disabilities
- On your life
- On society
- On inclusive sports

**Question 7.** How did you feel during the DDFI?

Elicitation questions:

a. What changes did you experience from preparation to DDFI in terms of experiencing “flow,” or being in the moment?

b. What factors affect your sense of engagement in an activity such as the DDFI?

c. What emotions did the DDFI evoke in you?

**Question 8.** Based on your experience, what is most important for a quality experience?

Elicitation questions:

a. What would an ideal experience look like?

b. How can a positive experience be maintained throughout the preparation for an event such as the DDFI?

c. If you had to do the process over again from the moment you first got involved, what would you have done differently? Why?

**Question 9.** Is there anything else you would like to tell me about the DDFI?
